# Near-infrared fluorescence imaging with intraoperative administration of indocyanine green for laparoscopic radical prostatectomy: Is it a useful weapon for pelvic lymph node dissection?

**DOI:** 10.1093/jscr/rjab614

**Published:** 2022-03-24

**Authors:** Raffaele Baio, Olivier Intilla, Umberto Di Mauro, Umberto Pane, Giovanni Molisso, Roberto Sanseverino

**Affiliations:** Department of Medicine and Surgery “Scuola Medica Salernitana”, University of Salerno, Baronissi, Salerno, Italy; Department of Urology, Umberto I Hospital, Nocera Inferiore, Salerno, Italy; Department of Urology, Umberto I Hospital, Nocera Inferiore, Salerno, Italy; Department of Urology, Umberto I Hospital, Nocera Inferiore, Salerno, Italy; Department of Urology, Umberto I Hospital, Nocera Inferiore, Salerno, Italy; Department of Urology, Umberto I Hospital, Nocera Inferiore, Salerno, Italy

**Keywords:** prostate cancer, bilateral pelvic lymph node dissection, indocyanine green

## Abstract

Near-infrared fluorescence imaging with indocyanine green has emerging applications in urologic surgery. This technology is strongly used in robotic surgery for several ablative and reconstructive procedures. On the contrary, it is not used at all in the urological laparoscopic surgery. To date, bilateral pelvic lymph node dissection represents the most accurate and reliable staging procedure for the detection of lymph node invasion in prostate cancer and bladder cancer. However, it is not devoid of complications. In this field, indocyanine green fluorescence-guided sentinel lymph node identification is an emerging technique, as accurate staging of urologic cancer could be enhanced by an intraoperative lymphatic mapping. Our goal was to show a high spatial resolution, real-time intraoperative imaging technique to recognize the main lymphatic drainage networks, avoiding at same time lymphatic vessel damage. Furthermore, the use of such an imaging system represents an absolute novelty in the field of urological laparoscopy.

## INTRODUCTION

Metastases account for most deaths due to malignancy [1], and the first site of metastases is usually the regional lymph nodes. Detection of lymph node invasion (LNI) is of major prognostic significance for many cancers [2, 3], especially for prostate and bladder cancers for which, at present, bilateral pelvic lymph node dissection (PLND) represents the most accurate and reliable staging procedure for the detection of LNI [4–7]. However, PLND is associated with increased operative time and is not devoid from severe complications. These limitations underscore the necessity of improving the identification of the primary lymph node pathway. In the last decade, optical imaging using near-infrared (NIR) fluorescence had emerged as a safe technique to visualize structures in real-time during surgery. Indocyanine green (ICG), an innocuous imaging dye, can be used for this scope [8]. In fact, it fluoresces bright green when viewed under NIR light (700–1000 nm). Currently, ICG NIR fluorescence-guided sentinel lymph node dissection (SLND) had emerged as a valid technique for the detection of regional LN for numerous tumors [8]. For urologic cancer, ICG NIR fluorescence-guided SLND is at its infancy but, recently, some authors reported that this technique is a promising complementary tool for lymphatic vessel visualization [9]. The goal of this case report is to demonstrate that ICG real-time fluorescent lymphography is a safe and feasible technique that can be used to avoid or enable early recognition of damage to the deep lymphatic vessels. This technique could significantly reduce post-operative complications related to the lymphatic system during laparoscopic radical prostatectomy.

## CASE REPORT

**Figure 1:**
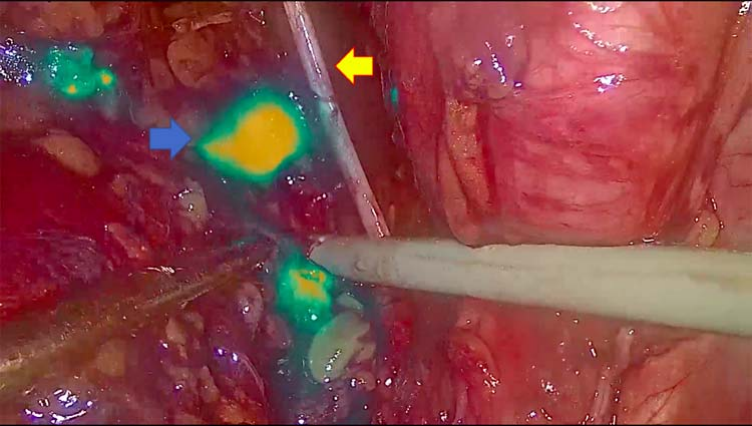
The blue arrow shows the lymph nodes which, due to the effect of indocyanine, take on a fluorescent green color. The yellow arrow indicates the obturator nerve.

**Figure 2:**
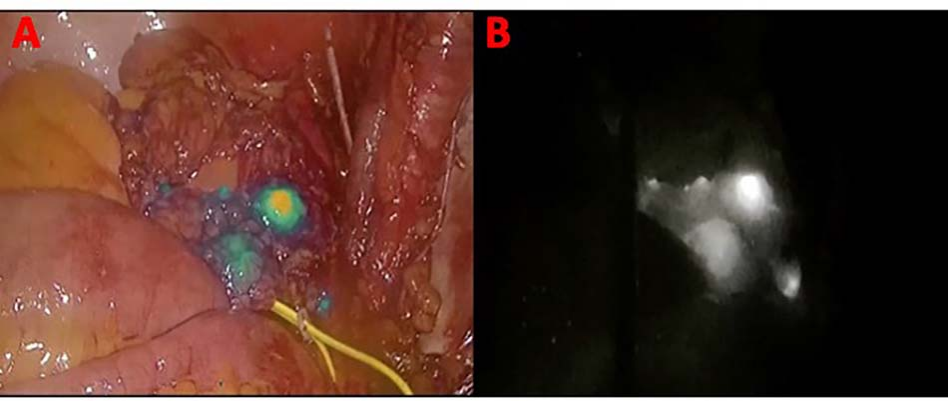
Panel (**A**) shows the lymph nodes in green color when the blue light is used. Panel (**B**) shows the lymph nodes in white when the ‘overlay’ function is used.

In November 2020, for a diagnosis of prostate cancer at prostatic biopsy, we intraoperatively performed ICG fluorescence-guided lymphography during a laparoscopic radical prostatectomy with pelvic lymphadenectomy. ICG was injected in the prostatic tissue of the patient transrectally through ultrasound identification of the gland. A fine needle was used connected to a 10 cc syringe, taking care to aspirate before injecting the tracer in order to avoid blood vessels. A dedicated laparoscopic high-definition camera system, provided by Karl Storz, was used in our case. This system allowed the surgeon to easily switch from White Light (WL) mode to ICG mode. For this reason, it was very simple to compare WL and ICG mode images. Furthermore, this technique is inexpensive, requiring only a small dose of ICG. Therefore, soon after ICG injection, the lymphatic vessels were identified in the pelvic cavity as fluorescent linear structures running side by side to the iliac vessels. Figure 1 shows the fluorescent lymph nodes in the obturator fossa. Then, using the ‘intensity map’ function (also called ‘overlay function’), lymph nodes can be seen as white structures as this function uses WL (instead of blue light) and eliminates the colors, as shown in Fig. 2. Surgical dissection was therefore performed, avoiding iatrogenic damage to major lymphatic structures. On histological examination, the prostate gland was found to be affected by an acinar adenocarcinoma (with Gleason Score 8, 4 + 4) involving ~20% of the right lobe and 35% of the left lobe. Twenty-four lymph nodes sent as obturators (15 on the right and 9 on the left side) and 10 lymph nodes sent as external iliacs (6 on the right and 4 on the left side) were free from metastases. The patient was discharged 5 days after surgery, and 6 months after surgery, he did not show any complications related to lymph node dissection.

## DISCUSSION

ICG has been used for decades in several medical applications such as retinal angiography, liver clearance tests (to measure hepatic function) and cardiac output monitoring [10]. Most recently, this technology has been used in different surgical fields, especially when a robotic approach is chosen. Our case is one of the very rare reports of applying ICG real-time fluorescent lymphography during laparoscopic radical prostatectomy. To our knowledge, there are no other reports in which this innovative technique is documented during laparoscopy in urology with the aid of videos. However, our experience is preliminary and limited to a single cases but it can be the first step to develop a simple, easy and low-risk technique for lymphatic sparing surgery. Obviously, further large series are required to define surgery outcomes and lymphatic complication rate. Many different surgical procedures can damage the lymph nodes and lymph vessels, leading to serious complications such as lasting lymphorrhea, lymphocele and lymphedema. Lymphorrhea is a persistent lymph loss that can occur from surgical drain in cases of intraperitoneal or retroperitoneal procedures or from surgical wounds in cases of superficial site surgery [11]. Lymphocele is a lymph-filled collection without an epithelial lining, most commonly located in the retroperitoneal space. The incidence and frequency of this type of complication constitute a serious clinical problem, leading to impaired postoperative wound healing, wound infection and abscess formation; this scenario results in a prolonged hospital stay and increased cost of treatment. When lymph nodes have been removed or damaged during surgical procedures, lymph is not well drained from the affected area. So, when the lymph is overcollected, especially in the arms and legs, the result is the swelling that is characteristic of lymphedema. This condition makes the affected arm or leg particularly vulnerable to infections such as cellulitis and lymphangitis and, more rarely, to lymphangiosarcoma. Effective treatment of these complications can be challenging and time-consuming [12, 13]. All this is to be taken into serious consideration especially for patients with prostate cancer for whom iliac–obturator lymphadenectomy during radical prostatectomy represents the most accurate method of staging the disease from a lymph node point of view. We think that lymphatic vessel sparing and prompt recognition of lymphatic structure damage, during laparoscopic radical prostatectomy, could lead to a reduction in the post-surgical complications rate and, consequently, a reduced hospital stay. In conclusion, although it is not necessary to perform fluorescence-enhanced surgery in all cases, we find the utilization of ICG-NIRF in lymph node dissection for prostate cancer useful. In effect, this technology in prostate cancer has a high detection rate, although its specificity to predict LN invasion remains poor. In our case, the pathological examination did not demonstrate an involvement of the pelvic lymph nodes; however, the use of such an imaging system has allowed us to remove the main lymphatic networks involved in the drainage of the gland (thus ensuring an accurate staging of the disease), with the possibility at the same time to recognize any serious damage to the lymphatic vessels during dissection.

## Supplementary Material

Video_for_Manuscript_rjab614

## References

[1] Mehlen P, Puisieux A. Metastasis: a question of life or death. Nat Rev Cancer 2006;6:449–58. 16723991 10.1038/nrc1886

[2] Nguyen DX, Bos PD, Massagué J. Metastasis: from dissemination to organ-specific colonization. Nat Rev Cancer 2009;9:274–84. 19308067 10.1038/nrc2622

[3] Pini G, Matin SF, Suardi N, Desai M, Gill I, Porter J, et al. Robot assisted lymphadenectomy in urology: pelvic, retroperitoneal and inguinal. Minerva Urol Nefrol 2017;69:38–55. 28009144 10.23736/S0393-2249.16.02823-X PMC9134864

[4] Heidenreich A, Pfister D. Pelvic lymphadenectomy in clinically localised prostate cancer: counting lymph nodes or dissecting primary landing zones of the prostate? Eur Urol 2014;66:447–9. 23954089 10.1016/j.eururo.2013.07.035

[5] Heidenreich A, Bastian PJ, Bellmunt J, Bolla M, Joniau S, van der Kwast T, et al. EAU guidelines on prostate cancer. Part 1: screening, diagnosis, and local treatment with curative intent-update 2013. Eur Urol 2014;65:124–37. 24207135 10.1016/j.eururo.2013.09.046

[6] Witjes JA, Compérat E, Cowan NC, De Santis M, Gakis G, Lebret T, et al. EAU guidelines on muscle-invasive and metastatic bladder cancer: summary of the 2013 guidelines. Eur Urol 2014;65:778–92. 24373477 10.1016/j.eururo.2013.11.046

[7] Mistretta FA, Boeri L, Grasso AA, Lo Russo V, Albo G, DE Lorenzis E, Maggioni M, Palmisano F, Dell ’orto P, Bosari S, Rocco B. Extended versus standard pelvic lymphadenectomy during robotassisted radical prostatectomy: the role of extended template as an independent predictor of lymph node invasion with comparable morbidity burden. Minerva Urol Nefrol 2017;69:475–85. 10.23736/S0393-2249.17.02838-7 28281741

[8] Polom K, Murawa D, Rho YS, Nowaczyk P, Hünerbein M, Murawa P. Current trends and emerging future of indocyanine green usage in surgery and oncology: a literature review. Cancer 2011;117:4812–22. 21484779 10.1002/cncr.26087

[9] Alander JT, Kaartinen A, Laakso A, Pätilä T, Spillmann T, Tuchin VV, et al. A review of indocyanine green fluorescent imaging in surgery. Int J Biomed Imaging 2012;2012:940585. 22577366 10.1155/2012/940585 PMC3346977

[10] Kamisaka K, Yatsuji Y, Yamada H, Kameda H. The binding of indocyanine green and other organic anions to serum proteins in liver diseases. Clin Chim Acta 1974;53:255e64. 4366883 10.1016/0009-8981(74)90107-7

[11] Metcalf KS, Peel KR. Lymphocele. Ann R Coll Surg Engl 1993;75:387e92. 8285540 PMC2498000

[12] Mori N . Clinical and experimental studies on the so-called lymphocyst which develops after radical hysterectomy in cancer of the uterine cervix. J Jpn Obstet Gynecol Soc 1955;2:178e203. 13286539

[13] White M, Mueller PR, Ferrucci JT, Butch RJ, Simeone JF, Neff CC, et al. Percutaneous drainage of postoperative abdominal and pelvic lymphoceles. AJR Am J Roentgenol 1985;145:1065e9. 3901705 10.2214/ajr.145.5.1065

